# A novel hydrolase with a pro-death activity from the protozoan parasite *Leishmania major*

**DOI:** 10.1038/s41420-019-0178-2

**Published:** 2019-05-24

**Authors:** Louise Basmaciyan, Pauline Jacquet, Nadine Azas, Magali Casanova

**Affiliations:** 1 UMR PAM A, Valmis team, 2 rue Angélique Ducoudray, BP 37013, 2107 Dijon Cedex, France; 2 Aix Marseille Univ, IRD, AP-HM, MEPHI, Marseille, France; 3 IHU-Méditerranée Infection, Marseille, France; 4 Aix Marseille Univ, IRD, AP-HM, SSA, VITROME, Marseille, France

**Keywords:** Apoptosis, Parasitic infection

## Abstract

Apoptosis is a cell death process generally described as involving a cascade of caspase activation, death receptors and/or pro- and antiapoptotic molecules from the BcL-2 family. But about 20 years ago, a caspase-independent apoptotic pathway has been described. Regarding this pathway, we can learn a lot from *Leishmania* parasites. Indeed, these parasitic protozoa enter, in response to different stimuli, in a form of cell death phenotypically similar to mammalian apoptosis but without involving caspases or death receptors. So far, only two proteins have been clearly identified as being involved in *Leishmania*-regulated cell death: the metacaspase and the endonuclease G. We report here the identification of a new protein modeled as a potential hydrolase, highly conserved among *Leishmania* species and absent in the very close parasite *Trypanosoma brucei*. This protein is involved in *L. major*-regulated cell death induced by curcumin, miltefosine and pentamidine, after gene overexpression and/or protein translocation to the nucleus. The identification of proteins involved in *Leishmania*-regulated cell death will provide a better understanding of nonconventional apoptotic pathways in higher eukaryotes. It will also allow the development of new therapeutic tools via the identification of new specific targets.

## Introduction

In multicellular organisms, the control suicide of cells is well established for eliminating superfluous cells during development of the organism or to remove damaged cells that might compromise fitness of the whole organism (reviewed in Elmore^1^). In unicellular organisms, since control suicide results in death of the whole organism, this phenomenon has long been a matter of debate. However, a population of unicellular organisms, such as yeasts or protozoa, should be considered as a group of communicating individuals whose goal is to ensure the entire population fitness to continue the cell cycle and not isolated cells that do not interact with each other. Thus, the death of unfit or damaged unicellular organisms can promote the survival of the whole population, as reviewed in Carmona-Gutierrez et al. for the yeast^2^. We can note that programmed cell death encompasses a physiological death. Therefore, in our study where apoptotic drugs are added, it is better to speak of regulated cell death, a “form of cell death that results from the activation of one or more signal transduction modules, and hence can be pharmacologically or genetically modulated”^3^. Furthermore, since apoptosis encompasses a form of cell death described on its morphology (cell rounding up, DNA fragmentation, plasma membrane modifications with maintenance of its integrity, etc.)^4^, and that these morphological changes can be observed during the cell death of many unicellular organisms^2,5^, we can speak not only of regulated cell death but also of apoptosis.

*Leishmania* are parasitic protozoa that are the causative agents of leishmaniases, neglected tropical diseases that threaten between 700,000 and 1 million people each year in about 97 countries (Global Health Observatory data from the World Health Organization, July 25, 2018). These parasites are transmitted to humans by the bite of an insect vector, the sand fly. In the insect, parasites proliferate as free-living flagellated forms called procyclic promastigotes within the midgut before differentiating into virulent metacyclic promastigotes and migrating to the proboscis^6,7^. In the mammalian host, promastigotes are taken up by macrophages where they transform into amastigotes. *Leishmania* parasites are called ancestral eukaryotes since they are highly distant phylogenetically from the traditionally studied organisms such as yeasts and mammals^8^. This high distance is underlined by several originalities. For instance, at the cellular level, *Leishmania* are flagellated protozoa covered by a sub-pellicular corset of microtubules, a dense helicoidal network of microtubules along the long axis of the cell^9^. At the molecular level, we can cite the high genomic plasticity or the mosaic aneuploidy^10^. Regarding cell death, several originalities also appear. While *Leishmania* cell death is phenotypically similar to mammal apoptosis, no death receptor, nor caspases, the key mammalian enzymes, are found bio-informatically. And the presence of members of the BcL-2 family is still a matter of debate^11^. Currently, only two proteins have been clearly identified as being involved in *Leishmania* cell death: (i) the metacaspase, which is a cysteine peptidase with a caspase-like histidine-cysteine catalytic dyad^12,13^ but with different substrate specificity than caspases^14^. We previously showed that the *L. major* metacaspase LmjMCA plays a role similar as the one of caspases in cell death^15^. This function is mediated by the catalytic domain released after the protein processing and also by the C-terminal domain through interaction with proteins involved in stress regulation or regulated cell death like the calpain-like cysteine peptidase^15^. (ii) The endonuclease G has also been identified, a nuclease with high structural and functional similarity with endonucleases G from higher eukaryotes^16,17^. After induction of cell death, the *Leishmania* EndoG, which is localized in the mitochondrion, migrates to the nucleus where it degrades double-stranded DNA, a characteristic feature of apoptosis^16,17^. Due to the numerous originalities of *Leishmania* parasites in comparison to classically studied organisms, the study of this ancestral eukaryote could shine a light on original pathways, notably nonconventional cell death pathways. This is of particular interest since many features remain to be elucidated concerning cell death^1^.

In this article, we studied a potential hydrolase highly conserved among *Leishmania* species and absent from the closely related parasite *Trypanosoma brucei*. This protein was involved in *L. major* cell death induced by several drugs (curcumin, miltefosine and pentamidine), through gene overexpression and/or protein translocation to the nucleus. Thus, this study allowed the identification of a new protein never identified in cell death, neither of *Leishmania* nor of mammals, giving a new insight in parasite cell death but also in cell death in general.

## Results

### LmjF.36.6540 is a potential hydrolase, whose sequence is conserved among *Leishmania* species

**Fig. 1:**
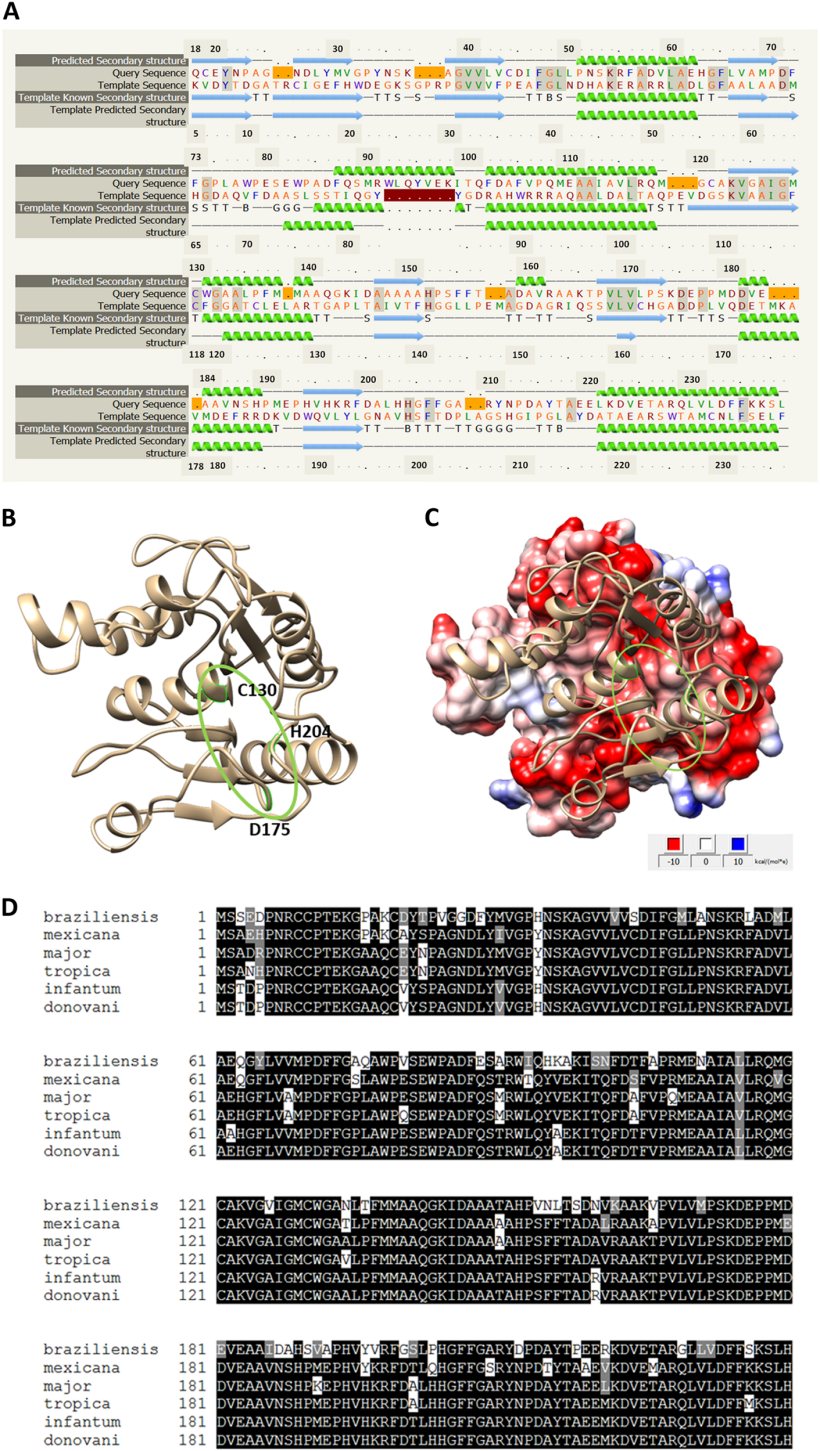
Sequence and predicted structure of LmjF.36.6540. **a** Alignment of the LmjF.36.6540 protein sequence and the PDB ID 4zi5, which is the crystal structure of dienelactone hydrolase-like promiscuous2 phospotriesterase p91 from metagenomic libraries, as done with the software Phyre2 (www.sbg.bio.ic.ac.uk/phyre2). The red stars indicate the three catalytic amino acids: C130, H204 and D175. **b** Tertiary structure of LmjF.36.6540 done with the UCSF Chimera software from the structural model from Phyre2. The catalytic triad is indicated in green. **c** LmjF.36.6540 electrostatic potential visualization: red for negative, white for neutral and blue for positive potentials (UCSF Chimera software, coulombic surface coloring). The catalytic triad is circled in green, highlighting the negative potential of the surface near the triad. **d** Alignment of the amino-acid sequences of the LmjF.36.6540 orthologs in different *Leishmania* species

The gene *LmjF.36.6540* encodes a protein of 240 amino acids. Thanks to the software Phyre2 (www.sbg.bio.ic.ac.uk/phyre2), 90% of the sequence has been modeled with 100% confidence by the single highest scoring template: the dienelactone hydrolase (Protein Data Bank (PDB) ID 4zi5). The amino acids form six helices and eight strands of β-sheet (Fig. 1a, b). The dienelactone hydrolase possesses characteristics of α/β-hydrolases: a catalytic triad consisting of a cysteine (Cys123), a histidine (His202) and an aspartic acid (Asp171)^18^. These residues are conserved at positions C126, H201 and D174 in PDB 4zi5. In LmjF.36.6540, as shown in Fig. 1a by a red star, these residues are also conserved: C130, H204 and D175, and are close to each other in the tertiary structure (Fig. 1b), which suggests preservation of protein function. As the model 4zi5 is also annotated as a phosphotriesterase in PDB, we tested this activity on LmjF.36.6540. Supplemental Fig. S1 shows that no phosphotriesterase activity could be detected for LmjF.36.6540. In the TriTrypDB database, this protein is annotated as similar to the endo-1-like protein (http://tritrypdb.org/tritrypdb/app/record/gene/LmjF.36.6540). However, when having a more detailed look at the surface charges, no positive patch that would bind DNA was identified. On the contrary, the surface of LmjF.36.6540 is generally negative, especially where the catalytic triad is localized (Fig. 1c). When analyzed with the PsortII prediction software (https://psort.hgc.jp/form2.html), a mitochondrial localization is predicted at 39.1%, a cytoplasmic localization at 26.1% and a nuclear localization at 21.7%, and no N-terminal signal peptide was found. Therefore, no clear cellular localization can be deduced from the predictions. This protein sequence is highly conserved among *Leishmania* species as shown in Fig. 1d, with percentage of identity from 72 to 99%. However, no homolog could be found in *T. brucei*, another protozoan parasite of the same *Trypanosomatidae* family.

### The *LmjF.36.6540* gene is overexpressed during cell death induced by curcumin and miltefosine

**Fig. 2:**
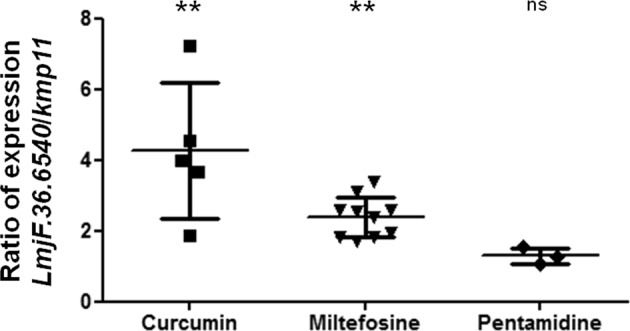
The *LmjF.36.6540* gene is overexpressed during cell death induced by curcumin and miltefosine. Ratio of *LmjF.36.6540*/*kmp11* expression, *kmp11* being a housekeeping gene used as a control, measured by RT-qPCR (mean value ± SD). Both *LmjF.36.6540* and *kmp11* expression in the apoptotic conditions were normalized to their expression in nontreated control cells. The treated conditions are: curcumin 50 µM for 24 h, miltefosine 40 µM for 24 h and pentamidine 100 µM for 24 h. For the statistical test, the *LmjF.36.6540* increase was compared to the *kmp11* increase with an unpaired Wilcoxon−Mann−Whitney test: n.s. not significant, **p < 0.01

In order to determine whether the expression of the *LmjF.36.6540* gene was modified under cell death conditions in *Leishmania*, we monitored its expression by reverse transcription quantitative PCR (RT-qPCR) after the addition of different proapoptotic molecules: curcumin, miltefosine and pentamidine^5^. We have chosen these three molecules because they induce three different apoptotic pathways as previously shown^19^. While miltefosine activates the metacaspase LmjMCA, curcumin induces *L. major* apoptosis through LmjMCA inhibition and pentamidine does not involve LmjMCA^19^. As shown in Fig. 2, the curcumin and miltefosine drugs induced overexpression of the *LmjF.36.6540* gene, the expression of this gene being 4.1 and 2.2 times higher on average than the expression of the housekeeping gene *kmp11* (Kinetoplastid Membrane Protein), respectively. This suggests the involvement of *LmjF.36.6540* in *Leishmania* cell death. On the contrary, the expression of the *LmjF.36.6540* gene was not clearly modified in pentamidine-treated cells (Fig. 2).

### Overexpression of *LmjF.36.6540* increases curcumin- and pentamidine-induced cell death

**Fig. 3:**
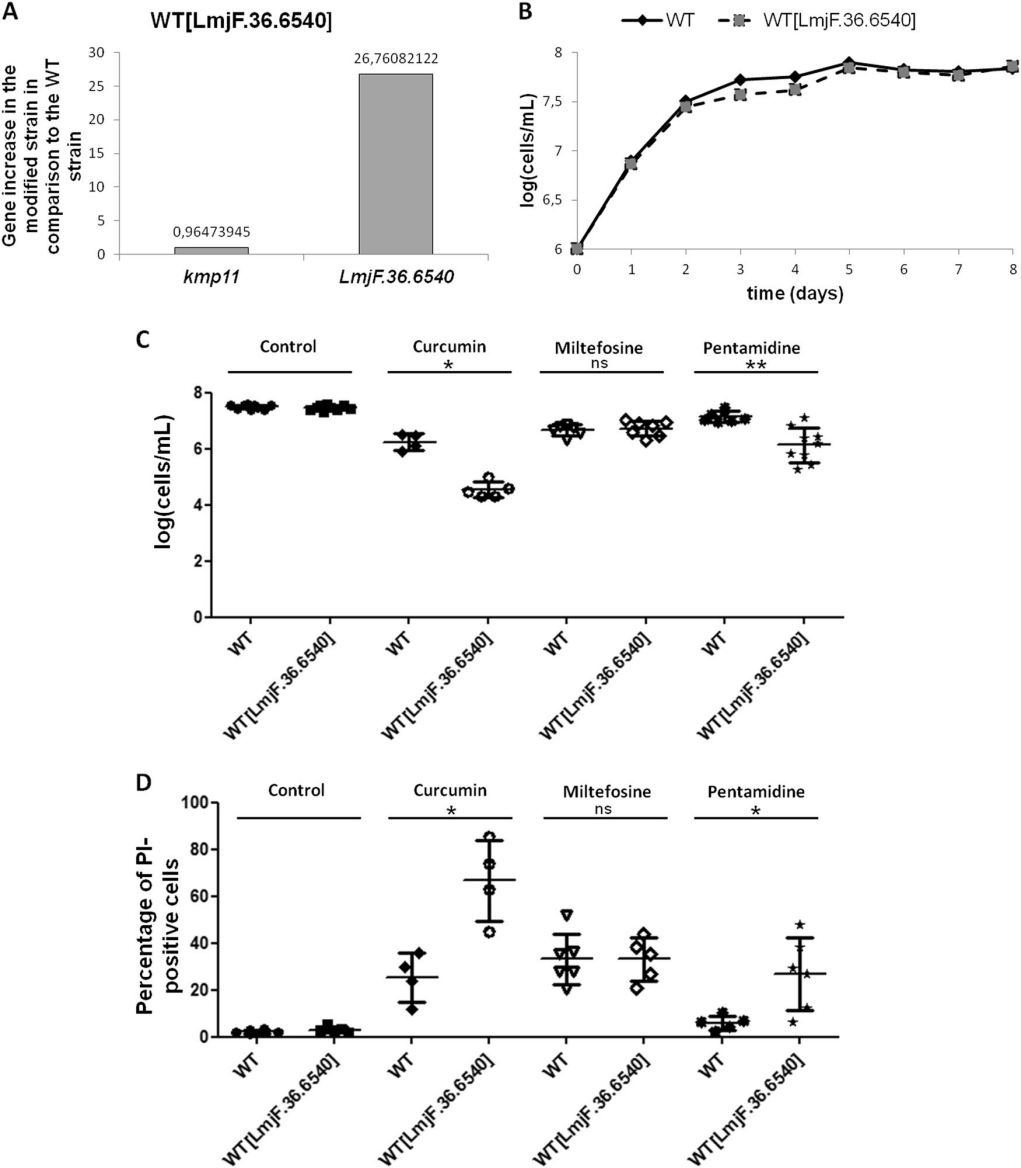
Overexpression of *LmjF.36.6540* increases curcumin- and pentamidine-induced cell death. **a** RT-qPCR quantification of *kmp11* and *LmjF.36.6540* in the *LmjF.36.*6540-overexpressing strain (WT[LmjF.36.6540]), in comparison to the WT strain. The value of gene expression is indicated on the graph. **b** Growth curves of the WT and *LmjF.36.6540*-overexpressing (WT[LmjF.36.6540]) strains: *n* ≥ 3. **c** Cell concentration of the WT and the *LmjF.36.6540*-overexpressing strain after a 24 h incubation without any drug (control) (*n* = 9), with 30 µM curcumin (*n* = 4/5), 40 µM miltefosine (*n* = 6/7) and 50 µM pentamidine (*n* = 7/9) (mean ± SD). **d** Percentage of PI-positive cells of the WT and *LmjF.36.6540*-overexpressing (WT[LmjF.36.6540]) strains after a 24 h incubation without any drug (control), with 30 µM curcumin, 40 µM miltefosine and 50 µM pentamidine (mean ± SD). Unpaired Wilcoxon−Mann−Whitney test: n.s. not significant, *p < 0.05, **p < 0.01

To better understand the role of LmjF.36.6540 in *L. major* cell death, the *LmjF.36.6540* gene was introduced into the expression vector pTH6nGFPc (cf. Fig. S2A). After transfection into *L. major*, the vector was maintained episomally and allowed the constitutive expression of the LmjF.36.6540 protein fused in C-terminal to the green fluorescent protein (GFP). We confirmed the overexpression of the gene by RT-qPCR: the *LmjF.36.6540* gene was 27.7 times more expressed in the modified strain than in the WT strain when the expression of the housekeeping gene *kmp11* was normalized to 1 (Fig. 3a). The modified strain had the same growth curve than the WT strain, as shown in Fig. 3b.

In a second stage, we investigated the consequences of *LmjF.36.6540* overexpression on *L. major* cell death. To that end, we cultivated the *LmjF.36.6540*-overexpressing strain with different proapoptotic molecules. When the proapoptotic drugs curcumin and pentamidine were added, the concentration of moving cells was significantly lower in the *LmjF.36.6540*-overexpressing strain than in the WT strain (Fig. 3c). On the contrary, the addition of miltefosine induced no difference in terms of concentration of moving cells (Fig. 3c). When evaluating the percentage of propidium iodide (PI)-positive cells, we correlated the decreased cell concentration in the overexpressing strain after the addition of curcumin and pentamidine with a significant PI-staining increase (Fig. 3d). Miltefosine induced no significant change in the percentage of PI-positive cells when *LmjF.36.6540* was overexpressed (Fig. 3d), which is in agreement with the absence of growth difference with this drug (Fig. 3c). Since the plasmid used to overexpress *LmjF.36.6540* introduced the *LmjF.36.6540* gene fused at its 3′-end to the GFP sequence, we could not use a green fluorescent molecule like calcein to define the type of cell death. We can only conclude that *LmjF.36.6540* overexpression induced more cell death in curcumin- and pentamidine-treated cells.

### Inhibition of *LmjF.36.6540* inhibits pentamidine-induced apoptosis

**Fig. 4:**
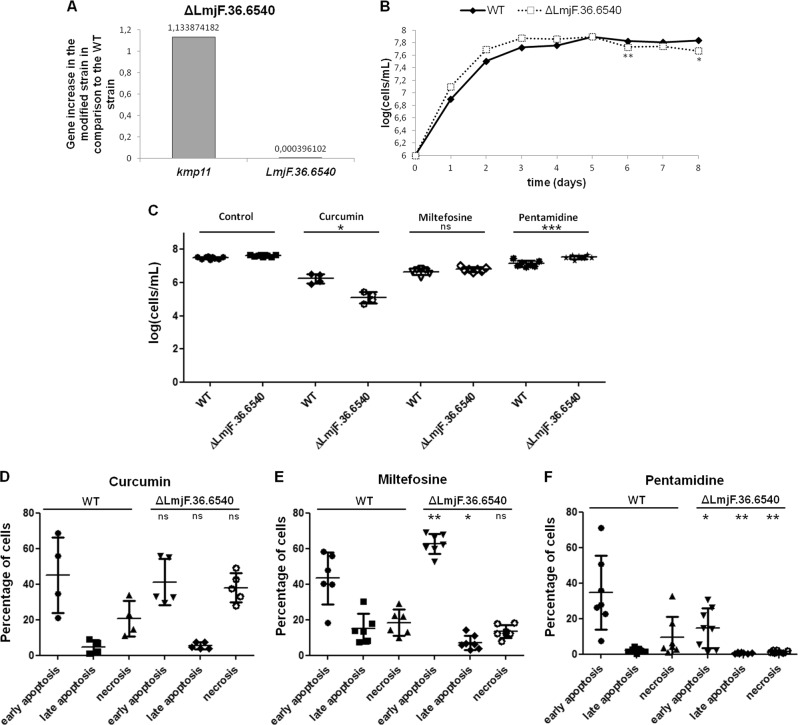
Inhibition of *LmjF.36.6540* inhibits pentamidine-induced apoptosis. **a** RT-qPCR quantification of *kmp11* and *LmjF.36.6540* in the *LmjF.36.6540-*deleted strain (Δ*LmjF.36.6540)*, in comparison to the WT strain. The value of gene expression is indicated on the graph. **b** Growth curves of the WT and *LmjF.36.6540*-deleted (Δ*LmjF.36.6540*) strains: *n* ≥ 3, except for Δ*LmjF.36.6540* at day 5 where *n* = 2. **c** Cell concentration of the WT and the *LmjF.36.6540*-deleted strain (Δ*LmjF.36.6540*) after a 24 h incubation without any drug (control) (*n* = 9), with 30 µM curcumin (*n* = 4), 40 µM miltefosine (*n* = 6/7) and 50 µM pentamidine (*n* = 7/8) (mean ± SD). **d–f** Percentage of cells of the WT and *LmjF.36.6540*-deleted (Δ*LmjF.36.6540*) strains in early apoptosis (calcein+/PI−), in late apoptosis (calcein+/PI+) and in necrosis (calcein−/PI+), as evaluated by flow cytometry after a 24 h incubation with 30 µM curcumin (**d**), 40 µM miltefosine (**e**) and 50 µM pentamidine (**f**) (mean ± SD). Unpaired Wilcoxon−Mann−Whitney test: n.s. not significant, *p < 0.05, **p < 0.01, ***p < 0.001

To confirm and better understand the role of Lmf.36.6540 in *L. major* cell death, we constructed, by CRISPR/Cas9, a strain in which the corresponding gene was deleted, using the method developed by Beneke et al.^20^ (Fig. S2B). We confirmed the inhibition of the expression of *LmjF.36.6540* by RT-qPCR (Fig. 4a) and by a standard PCR (Fig. S3). As shown in Fig. 4b, no difference was observed between the growth curve of the WT and the deleted strains, except at the end of the stationary phase (days 6 and 8), where the deleted strain grew much more slowly than the WT strain. This difference in growth could be related to the involvement of the protein in autophagy, as discussed later.

When the *LmjF.36.6540* gene was deleted, the proapoptotic molecule curcumin induced a lower concentration of moving cells, whereas pentamidine induced a significantly higher concentration than in the WT strain (Fig. 4c). No difference was observed between the WT and *LmjF.36.*6540-deleted strains after 24 h incubation with miltefosine, when counting the cells (Fig. 4c). In order to better understand the type of cell death involved in the *LmjF.36.6540*-deleted strain treated with different drugs, we monitored by flow cytometry the calcein- and PI-staining. Indeed, we previously showed that, in *Leishmania*, healthy cells appear calcein−/PI−, cells in early apoptosis are calcein+/PI−, cells in late apoptosis are calcein+/PI+ and cells in necrosis are calcein−/PI+^21^. We observed that the addition of curcumin had the same consequences in the WT strain than in the deleted strain (Fig. 4d), while miltefosine induced a higher percentage of early apoptotic cells but a lower percentage of late apoptotic cells (Fig. 4g). And pentamidine induced a significantly lower percentage of early and late apoptotic cells, as well as of necrotic cells, in the *LmjF.36.6540*-deleted strain in comparison to the WT strain (Fig. 4h). As a consequence, LmjF.36.6540 seems to be involved in miltefosine- and pentamidine-induced *L. major* cell death, but not in the same way. Pentamidine induced a decrease in cell death by apoptosis, while miltefosine induced contradictory events in the *LmjF.36.6540*-deleted strain: early apoptosis increase and late apoptosis decrease. These contradictory effects on apoptosis and necrosis could explain the absence of differences in the percentage of moving cells between the deleted and WT strains after the addition of miltefosine. Concerning curcumin, the difference in the concentration of moving cells was not correlated to modifications in the percentage of apoptotic or necrotic cells.

### Translocation of LmjF.36.6540 to the nucleus during parasite cell death

**Fig. 5:**
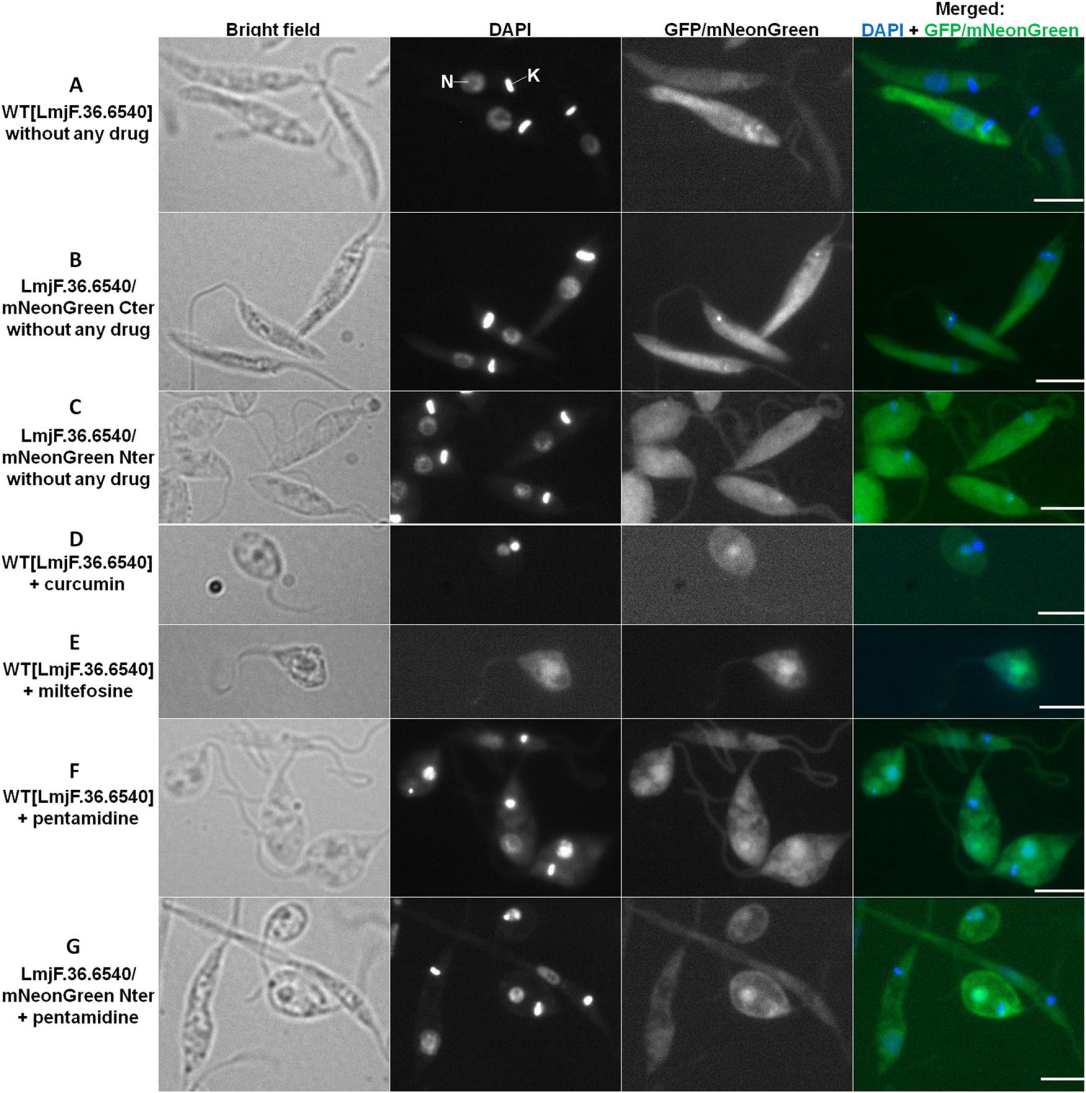
Translocation of LmjF.36.6540 to the nucleus during parasite cell death. Microscopical observation of different strains expressing a tagged version of LmjF.36.6540: bright field, DAPI, GFP or mNeonGren and DAPI/GFP or mNeonGren merged image (bar = 5 µm). The nucleus (N) and the mitochondrial DNA called kinetoplast (K) are indicated for a cell in the upper panel. Contrary to the elongated phenotype of control cells (**a**–**c**), the rounding of the cells after treatment with a proapoptotic drug (**d**–**g**) is a clear characteristic of apoptosis

Since the overexpression of *LmjF.36.6540* induced its fusion to the GFP, we could localize the corresponding protein in *Leishmania* cells by fluorescence microscopy. As shown in Fig. 5a, in the absence of cell death-inducing drugs, the LmjF.36.6540 protein was localized in the whole cell. It also localized as one (sometimes more) dot(s) usually at the anterior end of the cell. This dot was sometimes on the mitochondrial DNA called kinetoplast but it was often just anterior to the kinetoplast. As shown in Fig. 5b, c, we confirmed the localization of LmjF.36.6540 by tagging in situ the corresponding gene with the mNeonGreen sequence by CRISPR/Cas9 using the method developed by Beneke et al.^20^, the tag being, after *L. major* transfection, in C- or N-terminal of the protein (Fig. S2c, d, respectively). When the proapoptotic molecules curcumin, miltefosine and pentamidine were added, the *L. major* cells rounded, a characteristic feature of apoptosis as already described^22^ and, interestingly, LmjF.36.6540 translocated to the nucleus: Fig. 5d–f. This nuclear translocation of LmjF.36.6540 was not due to fusion with GFP since we obtained the same result with the in situ tagged protein (Fig. 5g). As a consequence, the activity of LmjF.36.6540 in *L. major* cell death could be linked to the nuclear translocation of the protein when a proapoptotic stimulus is added.

### Inhibition of LmjF.36.6540 induces a virulence increase

**Fig. 6:**
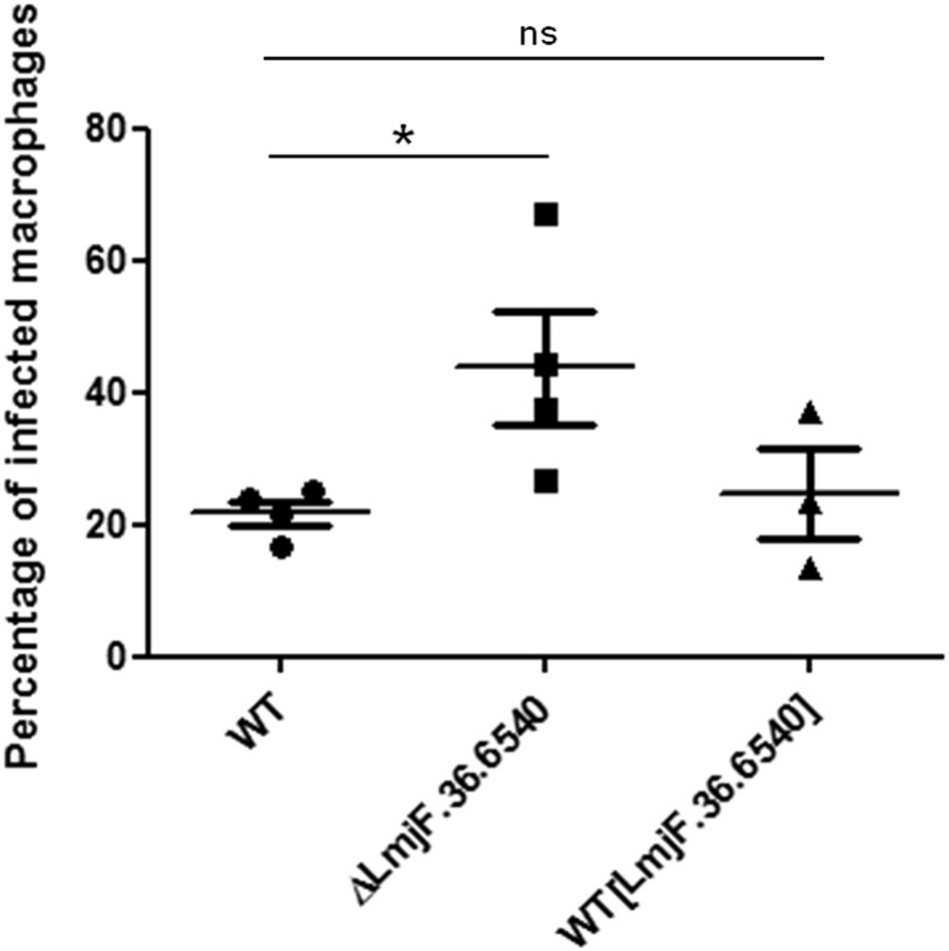
Deletion of *LmjF.36.6540* increases cell virulence. Percentage of infected macrophages (THP1) after infection with the WT, the *LmjF.36.6540*-deleted (Δ*LmjF.36.6540*) and *LmjF.36.6540*-overexpressing (WT[LmjF.36.6540]) strains (mean ± SD). Unpaired Wilcoxon−Mann−Whitney test: n.s. not significant, *p < 0.05

In order to have a first view of the role of LmjF.36.6540 in the mammalian host, we infected macrophages with either the *LmjF.36.6540*-deleted or the *LmjF.36.6540*-overexpressing strain. As shown in Fig. 6, the overexpression of LmjF.36.6540 had no consequences on the percentage of *L. major*-infected macrophages. On the contrary, *LmjF.36540* deletion induced a significantly higher percentage of infected macrophages. This reminds the consequences of *L. mexicana* metacaspase deletion^23^. As a consequence, LmjF.36.6540 is involved in *L. major* virulence, confirming its interest as a new therapeutic target.

## Discussion

LmjF.36.6540 encodes a protein with high similarity to the dienelactone hydrolase. The presence of the catalytic triad (amino acids C130, H204 and D175) and the localization of the catalytic amino acids in the tertiary structure suggest that the hydrolase function is conserved in LmjF.36.6540. To further our understanding of protein function, we tested its phosphotriesterase activity but to no avail. Furthermore, the LmjF.36.6540 seems to possess no DNA binding site which would appear positively charged in the tertiary structure. This result was confirmed when incubating the protein with plasmid DNA: no clear smear could be observed and the DNA was not retained in the wells during electrophoresis owing to its binding to the protein (Fig. S4). All these features are consistent with the absence of nuclease activity of LmjF.36.6540. Thus, the TriTrypDB annotation (“similarity to endo-1-like protein”) must be corrected and more experiments must be conducted to identify the LmjF.36.6540 physiological function.

In order to investigate the link between LmjF.36.6540 and *Leishmania*-regulated cell death, we tested three molecules described as proapoptotic in *L. major*^22^ and as inducing different proapoptotic pathways^19^: curcumin, miltefosine and pentamidine. The results obtained are summarized in Table 1. Curcumin induced the overexpression of the *LmjF.36.6540* gene, and the overexpression of this gene increased curcumin-induced *L. major* cell death. Thus, LmjF.36.6540 seems to be involved in *L. major* cell death pathway induced by curcumin, even if no difference in cell death was observed when the gene was deleted. Furthermore, LmjF.36.6540 was involved in the cell death pathway induced by miltefosine as shown by overexpression of the gene after the addition of the drug and by the consequences of gene deletion on *L. major* cell death. However, LmjF.36.6540 had contradictory consequences: deletion of the gene induced increased miltefosine-induced early apoptosis but decreased miltefosine-induced late apoptosis. These contradictory effects could explain the absence of modification of cell concentration after the addition of miltefosine, when LmjF.36.6540 was overexpressed or deleted, in comparison to WT cells. Finally, LmjF.36.6540 was undoubtedly involved in pentamidine-induced cell death: its gene deletion inhibited *L. major* pentamidine-induced cell death, while its gene overexpression increased the pentamidine-induced cell death. On the contrary, the LmjF.36.6540 protein does not appear to be involved in *L. major* cell death induced by H_2_O_2_. Indeed, the expression of the corresponding gene was not modified after the addition of H_2_O_2_ (Fig. S5A), and no significant difference was observed in terms of *L. major* cell death in the *LmjF.36.6540*-overexpressing (Fig. S5B) or deleted strains (Fig. S5C), compared to the WT strain. The addition of the different apoptotic drugs induced LmjF.36.6540 nuclear translocation but one can wonder whether this translocation was linked to apoptosis since H_2_O_2_ also induced LmjF.36.6540 nuclear translocation (Fig. S5D), while the protein does not seem to be involved in the cell death pathway induced by this molecule.

**Table 1:** Summary of the results obtained concerning the link between LmjF.36.6540 and cell death

	RT-qPCR	Overexpression	Deletion	Localization
Curcumin	++	**↗**cell death	n.d.	(nuclear translocation)
Miltefosine	++	n.d.	**↗** early apoptosis **↘** late apoptosis	(nuclear translocation)
Pentamidine	n.d.	**↗**cell death	early/late **↘** apoptosis/necrosis	nuclear translocation++

*n.d.* no difference, RT-qPCR reverse transcription quantitative PCR

**Fig. 7:**
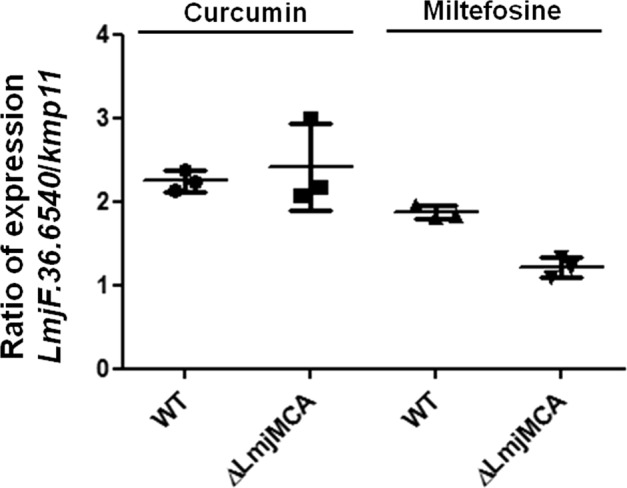
The *LmjF.36.6540* gene is not overexpressed in the LmjMCA-deleted strain after the addition of miltefosine. Ratio of *LmjF.36.6540*/*kmp11* expression, measured by RT-qPCR, in the presence of 30 µM of curcumin or 40 µM of miltefosine, in the WT and the LmjMCA-deleted (Δ*LmjMCA*) strains (mean ± SD). Both *LmjF.36.6540* and *kmp11* expression in the apoptotic conditions were normalized to their expression in nontreated control cells. While curcumin induces *LmjF.36.6540* overexpression, the *LmjMCA* gene being deleted or not, miltefosine induces *LmjF.36.6540* overexpression only in the presence of *LmjMCA*

**Fig. 8:**
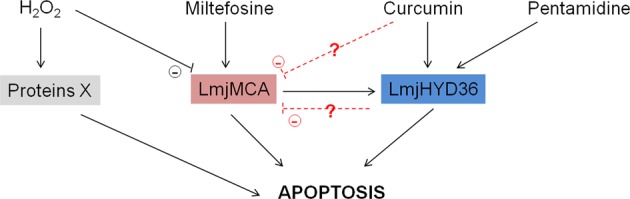
Model. In the model that we propose, H_2_O_2_ would induce apoptosis via the induction of other proteins (noted “proteins X” since unknown until now) than LmjMCA or LmjF.36.6540 (noted LmjHYD36). Miltefosine would induce LmjMCA activation, which would activate LmjHYD36, both proteins inducing *Leishmania* apoptosis. On the contrary, curcumin would activate only LmjHYD36, inducing apoptosis. Whether curcumin inhibits LmjMCA or whether curcumin has no direct action on LmjMCA remains to be elucidated. Finally, pentamidine would activate LmjHYD36 independently of LmjMCA, inducing *Leishmania* apoptosis. The relationship between LmjHYD36 and LmjMCA (whether LmjHYD36 inhibits LmjMCA or whether it has no action on LmjMCA) remains also to be elucidated

In a previous article, we demonstrated that *L. major* possesses no caspase, the key enzyme of mammalian apoptosis, but possesses a metacaspase called LmjMCA whose function is similar to that of caspases^15^. Indeed, LmjMCA has been shown to be involved in *L. major* cell death either by the release of its catalytic domain or by interaction of its C-terminal domain with partners involved in stress regulation or cell death^15^. In order to evaluate the link between LmjMCA and *LmjF.36.6540*, we carried out RT-qPCR experiments. We observed that while *LmjF.36.6540* expression was increased in the WT strain after the addition of miltefosine, the gene was not overexpressed in the LmjMCA-deleted strain after the addition of miltefosine (Fig. 7). As a consequence, LmjF.36.6540 seems to act downstream of LmjMCA in the miltefosine apoptotic pathway. On the contrary, the *LmjF.36.6540* gene was overexpressed after the addition of curcumin, in the WT strain as well as in the LmjMCA-deleted strain (Fig. 7). Therefore, curcumin seems to induce an apoptotic pathway in which either LmjF.36.6540 is upstream of LmjMCA or Lmj.36.6540 is independent of LmjMCA. Furthermore, regarding LmjMCA, we previously identified three apoptotic pathways: one activating LmjMCA, induced by miltefosine, one inhibiting LmjMCA, induced by curcumin and H_2_O_2_, and one that does not involve LmjMCA that is induced by pentamidine^19^. To better understand the link between LmjMCA and LmjF.36.6540 in the different apoptotosis pathways, we propose the model presented in Fig. 8. We propose that H_2_O_2_ induces apoptosis via the induction of other proteins than LmjMCA or LmjF.36.6540. These proteins, which are unknown until now, are noted “proteins X” in the model. Miltefosine would induce LmjMCA activation, which would activate LmjF.36.6540, both proteins inducing *Leishmania* apoptosis. On the contrary, curcumin would activate only LmjF.36.6540, inducing apoptosis. Whether curcumin inhibits LmjMCA or whether curcumin has no direct action on LmjMCA remains to be elucidated. Finally, pentamidine would activate LmjF.36.6540 independently of LmjMCA, inducing *Leishmania* apoptosis. The relationship between LmjF.36.6540 and LmjMCA (whether LmjF.36.6540 inhibits LmjMCA or whether it has no action on LmjMCA) remains also to be elucidated.

A study from McNicoll et al. showed that, during the promastigote to amastigote differentiation, the protein LmjF.36.6540 was highly overexpressed by mass spectrometry and the corresponding gene was highly overexpressed by microarray analysis^24^. Yet, *Leishmania* differentiation is characterized by autophagy, the cell survival process that allows cells to survive nutrient depletion^25,26^. This suggests that LmjF.36.6540 is involved in autophagy, which we confirmed by showing gene overexpression when cells were incubated without serum, an autophagy condition (Fig. S6). The role in autophagy could explain why the deleted strain grew slower at the end of the stationary phase (Fig. 4b). Indeed, autophagy appears during *Leishmania* differentiation from procyclic promastigotes to metacyclic promastigotes and at the end of the stationary phase^25^ and is characterized by growth defects^22^. The involvement of LmjF.36.6540 in *L. major* apoptosis and autophagy validates the close link between these two classically opposed processes already demonstrated in mammals^27^ and suggested in *Leishmania*^5^.

In conclusion, the study of an ancestral eukaryote allowed us to identify a new protein involved in *Leishmania* cell death that we propose to call LmjHYD36 for its hydrolase function and the location of the gene on chromosome 36. Until now, this protein had never been identified as being involved in cell death in any organism as ancestral as higher eukaryotes. This will complete the understanding of the parasite cell death, allowing in the future the targeting of the corresponding metabolic pathways to treat leishmaniases. It will also allow a better understanding of higher eukaryote cell death, through the discovery or a better understanding of unconventional apoptotic pathways.

## Materials and methods

### Parasites

*L. major* “Friedlin” promastigotes (MHOM/IL/81/Friedlin) were grown in Schneider’s *Drosophila* medium (Thermo Fisher Scientific, Waltham, MA, USA) supplemented with 100 U/mL penicillin, 100 µg/mL streptomycin, 2 mM glutamine and 20% fetal calf serum (Thermo Fisher Scientific) at 26° C.

### Structural analysis of LmjF.36.6540 and amino-acid sequence alignment

The three-dimensional structure of LmjF.36.6540 was obtained using the software Phyre2 (www.sbg.bio.ic.ac.uk/phyre2). With this structure, the electrostatic potential was obtained using the Coulombic surface coloring option from UCSF Chimera software^28^. The amino-acid sequences of the different *Leishmania* species were aligned with the Clustal Omega software (https://www.ebi.ac.uk/Tools/msa/clustalo/).

### Phosphotriesterase activity

LmjF.36.6540 and *Sso*Pox phosphotriesterase activities were determined with ethyl-paraoxon (1 mM, Sigma-Aldrich, Saint-Louis, MO, USA). Substrate degradation was followed at 405 nm with microplate reader (Synergy HT, BioTek, USA) and slopes (mOD/min) were obtained using Gen5.1 software. Experiment was performed in triplicate at 25° C for a reactional volume of 200 µL in 50 mM HEPES pH 8.0, 150 mM NaCl buffer.

### Cloning, expression and purification of LmjF.36.6540

The gene encoding LmjF.36.6540 was designed to include a Strep-tag at the N-terminus and optimized for *Escherichia coli* expression. It was synthetized by GenScript and ligated between the *Nde*I and *Not*I cut sites of a pET22b(+) plasmid. *E. coli* BL21(DE3)-pGro7/GroEL (TaKaRa, Saint-Germain-en-Laye, France) grown in ZYP-5052 media were used for the expression of the recombinant protein. When the culture reached an optical density of 0.8 at 600 nm at 37° C, the incubator temperature was changed to 18° C and l-arabinose (0.2% m/v) was added in order to induce chaperones expression. After 20 h, cells were harvested by centrifugation (5,000 × *g*, 30 min, 4° C) and the resulting pellet was resuspended in Wash Buffer (50 mM Tris pH 8, 300 mM NaCl) and stored at −80° C overnight. Frozen *E. coli* were thawed and incubated on ice for 1 h after adding lysozyme, DNAse I and phenylmethylsulfonyl fluoride to final concentrations of respectively 0.25 mg/mL, 10 µg/mL and 0.1 mM. Partially lysed cells were then disrupted by three consecutive cycles of sonication (30 s, amplitude 45) performed on a Q700 sonicator system (QSonica, Newtown, CT, USA). Cells debris were discarded following a centrifugation step (10,000 × *g*, 20 min, 4° C). The LmjF.36.6540 protein was purified with an ÄKTA avant system (GE Healthcare, Chicago, IL, USA) using Strep-tag affinity chromatography (Wash buffer: 50 mM Tris pH 8, 300 mM NaCl and Elution buffer: 50 mM Tris pH 8, 300 mM NaCl, 2.5 mM desthiobiotin) on a 5 mL StrepTrap HP column (GE Healthcare). Fractions containing the protein of interest were pooled. Protein purity was assessed using 12.5% SDS-PAGE analysis (Coomassie stain). Protein expression was confirmed by performing MALDI-TOF MS analysis on gel bands previously obtained by SDS-PAGE. Protein concentration was measured using a Nanodrop 2000c spectrophotometer (Thermo Fisher Scientific).

### Nuclease cleavage assay

For testing the nuclease activity of LmjF.36.6540, 100 or 200 ng of the linear plasmid DNA pTH6cGFPn, digested by the restriction enzymes *Hpa*I and *Mfe*I, were incubated for 1 h at 37° C with different concentrations of the purified LmjF.36.6540 protein in a final volume of 20 µL. The assay buffer was composed of 10 mM KCl, 3 mM MgCl_2_, 0.5 mM dithiothreitol, 20 mM HEPES, pH 7.5. Digested DNA was resolved on a 1% agarose gel, stained with SYBR Safe (Thermo Fisher Scientific) and visualized under UV light.

### *Leishmania* treatment

Logarithmic *L. major* cells were incubated with 1 µM amphotericin B (Sigma-Aldrich), curcumin (Sigma-Aldrich) at 50 µM for RT-qPCR experiments and 30 µM for the other experiments, 400 µM H_2_O_2_ (Sigma-Aldrich), 40 µM miltefosine (Santa Cruz Biotechnology, Dallas, TX, USA) or pentamidine (Sigma-Aldrich) at 100 µM for RT-qPCR or 50 µM for the other experiments. This was done for 24 h from 10^6^ cells/mL for the IC50 experiment or from 10^7^ cells/mL for all other experiments. The cells were counted after 24 h with a hemocytometer.

For nutrient deprivation, logarithmic *L. major* cells were harvested by centrifugation at 600 × *g* for 10 min, washed once with sterile PBS and incubated at 10^7^ cells/mL in a serum-deprived medium.

### Reverse transcription quantitative PCR

For RNA extraction, the RNeasy Plus mini kit was used (Qiagen, Courtaboeuf, France). Cells were harvested by centrifugation at 600 × *g* for 10 min and lysed with the RLT-Plus solution. After passing through a gDNA eliminator column, cells were washed with ethanol 70%, RW1 and RPE buffers. The concentration of the eluated RNAs was evaluated using a Nanodrop 2000c spectrophotometer (Thermo Fisher Scientific) before being aliquoted and conserved at −80° C. One-step reverse transcription was performed using the high-capacity cDNA reverse transcription kit (Applied Biosystems, Foster City, CA, USA). RNA (10 µL) was added to an equal volume of RT-PCR mix containing RT buffer, dNTPs, random primers and the multiscribe reverse transcriptase. Reverse transcription was performed using the following cycling conditions: 10 min at 25° C, 120 min at 37° C and 5 min at 85° C. For quantitative PCR, 5 µL of cDNA was added to 20 µL of PCR mix containing Sybr Green I (Roche, Meylan, France) and placed in a Light Cycler 480 with the following cycling conditions: Taq polymerase activation at 95° C for 10 min and 45 cycles of amplification of 15 s at 95° C and 60 s at 60° C. The *kmp11* (Kinetoplastid Membrane Protein 11) gene was used as control, having the same level of expression under all conditions used. Gene expression was calculated using the Pfaffl method: (eff_gene_)^ΔCtgene(control-treated)^ with “eff” the efficiency, “control” the control condition without any drug, and “treated” the death condition. The PCR efficiency of the different oligonucleotide pairs was determined using the serial dilution method on the basis of a linear regression slope. The oligonucleotides used in this study are listed in Table S1.

### Construction of the *LmjF.36.6540*-overexpressing strain

The *LmjF.36.6540* gene was PCR-amplified from *L. major* genomic DNA. The PCR product was cloned into pGEM-T-Easy (Promega, Madison, WI, USA) and then inserted into the expression vector pTH6nGFPc (kind gift from Patrick Bastien, Montpellier University) after digestion with *Mfe*I and *Hpa*I restriction enzymes, vector that places the GFP sequence at the 3′-end of the *LmjF.36.6540* sequence. After transfection, this vector was maintained episomally in *L. major* cells.

### Construction of the *LmjF.36.6540*-deleted and *LmjF.36.6540*-tagged strains

Deletion and tagging at the 5′ and 3′-end of the *LmjF.36.6540* gene were performed as described in the article of Beneke et al.^20^. The primers were designed thanks to the online tool developed by the authors: http://leishgedit.net/ (Suppl. Table 1). For the PCR-amplification of the targeting fragments of pT for KO or pPLOT (for tagging) cassettes (kind gift from Eva Gluenz, University of Oxford), 20 ng plasmid (LC100 and LC101 for the KO and LC106 for the taggings), 0.4 mM dNTP, 0.5 µM each of gene-specific forward and reverse primers and 0.5 µL of Phusion High-Fidelity DNA Polymerase (New England Biolabs, Ipswich, MA, USA) were mixed in 50 µL final. To amplify the guide RNAs, 0.6 mM dNTP, 2 µM each of gene-specific forward and reverse primers and 0.5 µL of Phusion High-Fidelity DNA Polymerase were mixed in 50 µL final. The PCR conditions were as follows: 30 s at 98° C, then 40 cycles of 10 s at 98° C, 30 s at 62° C and 1 min at 72° C, and a final elongation step of 10 min at 72° C. After assessing the presence of the expected PCR products by migration on an agarose gel, the PCR products (each PCR being done in duplicate) were pooled and purified with the Wizard SV Gel and PCR Clean-Up System (Promega). The purified PCR products were heat sterilized at 95° C for 5 min before use for transfection. For verification of the deletion of the *LmjF.36.6540* gene, after transfection, the genomic DNA was extracted with the EZ1 DNA tissue kit (Qiagen) and different PCR were carried out with the AmpliTaq Gold 360 Master Mix ((Thermo Fisher Scientific).

### Parasite transfection

*L. major* cells expressing Cas9 and T7 (kind gift from Eva Gluenz, University of Oxford) in logarithmic phase were transfected: either 10^7^ cells with 30 µg of purified PCR for the KO, or 3 × 10^6^ cells with 15 µg of PCR for the taggings. The transfection buffer was composed of 90 mM sodium phosphate, 5 mM potassium chloride, 0.15 mM calcium chloride, 50 mM HEPES, PH 7.3. The same transfection without purified PCR was used as a control. The transfections were performed in 2 mm gap cuvettes (Lonza, Bâle, Switzerland) with program X-001 of the Amaxa Nucleofector II (Lonza). Transfected cells were immediately transferred into 5 mL prewarmed medium and left to recover overnight at 26° C before adding Geneticin (Sigma-Aldrich) at 20 µg/mL and Puromycin (Sigma-Aldrich) at 30 µg/mL for the KO and 20 µg/mL Blasticidin (Sigma-Aldrich) for the taggings.

### Calcein and PI labeling

Cells were washed once in PBS and resuspended in 1 mL of calcein (LIVE/DEAD® Viability/Cytotoxicity Kit for mammalian cells, Molecular Probes, OR, USA) diluted 1/80 in DMSO and 5 µL PI at 0.5 mg/mL. The mixed sample was then incubated for 15–20 min at room temperature and protected from light. The cells were analyzed by flow cytometry using 488 nm excitation and measuring green fluorescence emission for calcein (530/30 bandpass) and red fluorescence emission for PI (610/20 bandpass) on the BD LSRFortessa™ cell analyzer. Data were exported and analyzed with Flowjo software for evaluation of the percentage of calcein- and PI-positive cells.

### Fluorescence microscopy

For the intracellular localization of LmjF.36.6540, fluorescent cells were fixed in 4% paraformaldehyde, washed in PBS, deposited on microscope fluorescence slides and air-dried. Slides were mounted with SlowFade Gold Antifade Mountant with DAPI (Thermo Fisher Scientific). Observations were made using a BX51 fluorescence microscope (Olympus, Rungis, France) and images were acquired using the fluorescence imaging system Cell^A^ (Olympus).

### Macrophage infectivity assay

THP1 monocytes at a concentration of 10^5^ cells/mL were differentiated in macrophages by the addition of 50 ng/mL phorbol myristate acetate. The differentiated THP1 were then infected in Labtek chamber slides by 10^6^ cells/mL stationary phase *L. major* promastigotes obtained after a 7-day culture from 10^6^ cells/mL and acidification at pH 5.6 the day before macrophage infection. Extracellular *Leishmania* parasites were washed five times 24 h post infection. At day 4 post infection, cells were fixed for 20 min at 4° C with 4% paraformaldehyde, washed in PBS and air-dried. The slides were mounted with SlowFade Gold Antifade Mountant with DAPI (Thermo Fisher Scientific). The percentage of infected macrophages was determined by examination of a minimum of 200 macrophages per well, in duplicate or triplicate, under fluorescence microscopy using a BX51 fluorescence microscope (Olympus) coupled with the fluorescence imaging system Cell^A^ (Olympus).

### Statistical analysis

For statistical analysis, unpaired Wilcoxon−Mann Whitney tests were performed with BioStaTGV. Results, obtained from a minimum of three independent experiments, were considered statistically significant when *p* < 0.05.

## Supplementary information

Legend of supplemental figures

Supplemental table S1

Supplemental figure S1

Supplemental figure S2

Supplemental figure S3

Supplemental figure S4

Supplemental figure S5

Supplemental figure S6
