# Supplementary material for: A novel hydrolase with a pro-death activity from the protozoan parasite Leishmania major
Source: Cell Death Discov. 2019 May 24;5:99. doi: 10.1038/s41420-019-0178-2 (PMC6534544; doi:10.1038/s41420-019-0178-2)
Supplement: Supplementary file 1 — Legend of supplemental figures [file 41420_2019_178_MOESM1_ESM.doc]

**SUPPLEMENTAL FIGURES**

**Figure S1. Phosphotriesterase activity.** LmjF.36.6540 and *Sso*Pox (used as a control) phosphotriesterase activities were determined with ethyl-paraoxon.

**Figure S2. Schematization of the different constructions done in this study. (A)** Insertion into the pTH6nGFPc plasmid of the *LmjF.36.6540* sequence, allowing, after *L. major* transfection, overexpression of LmjF.36.6540 and the episomal C-terminal GFP-tagging of the LmjF.36.6540 protein. HygroR = hygromycin resistance gene. **(B)** Deletion by CRISPR/Cas9 of the *LmjF.36.6540* gene replaced *in situ* by the geneticin or puromycin resistance genes from pT plasmids. To delete the target gene, two sgRNAs (symbolized by scissors) cut immediately upstream and downstream of the *LmjF.36.6540* gene. GeneticinR = geneticin resistance gene; PuromycinR = puromycin resistance gene. **(C)** *In situ* C-terminal mNeonGreen-tagging of LmjF.36.6540 by CRISPR/Cas9, after cutting in 3’ of the *LmjF.36.6540* gene thanks to a sgRNA. **(D)** *In situ* N-terminal mNeonGreen-tagging of LmjF.36.6540 by CRISPR/Cas9, after cutting in 5’ of the *LmjF.36.6540* gene thanks to a sgRNA.

**Figure S3. Localization of the different primers used for verification of the deleted construction and results of the corresponding PCR. (A)** Schematization of the localization of the primers used for PCR for verification of the correct deletion of *LmjF.36.6540*. The different primers are numbered from 1 to 8. **(B)** Agarose gel electrophoresis showing the results of the different PCR done for verification of the correct deletion of *LmjF.36.6540*. The pairs of primers used are indicated at the top of each column and the expected length of each amplicon at the bottom of each column.

**Figure S4. Nuclease activity assay.** Agarose gel electrophoresis of 100 ng or 200 ng of linear plasmid (pTH6cGFPn digested by HpaI and MfeI) incubated for 1 h at 37 °C with different concentrations of the LmjF.36.6540 protein.

**Figure S5. LmjF.36.6540 is not involved in *L. major* H_2_O_2_-induced cell death. (A)** Concentration of the WT and the *LmjF.36.6540*-overexpressing (WT[LmjF.36.6540]) strains after a 24 h incubation with 400 µM H_2_O_2_ (mean ± SD). **(B)** Percentage of PI-positive cells of the WT and *LmjF.36.6540*-overexpressing (WT[LmjF.36.6540]) strains after a 24 h incubation with 400 µM H_2_O_2_ (mean ± SD). **(C)** Percentage of cells of the WT and *LmjF.36.6540*-deleted (ΔLmjF.36.6540) strains in early apoptosis (calcein+/PI-), in late apoptosis (calcein+/PI+) and in necrosis (calcein-/PI+), as evaluated by flow cytometry after a 24 h incubation with 400 µM H_2_O_2_. In experiments from figures S5A, B and C, no significant difference was observed between the WT strain and the recombinant strain (unpaired Wilcoxon-Mann Whitney test). **(D)** Microscopical observation of *LmjF.36.6540*-overexpressing cells (WT[LmjF.36.6540]) incubated for 24 h with 400 µM H_2_O_2_ (bar = 5µm).

**Figure S6. The LmjF.36.6540 gene is overexpressed in autophagic conditions.** Ratio of *LmjF.36.6540*/*kmp11* expression, measured by RT-qPCR, when *L. major* cells were cultivated in a serum-deprived medium, gene expression in apoptotic conditions being normalized to the expression in non-treated control cells (mean ± SD).
