# Supplementary material for: A novel hydrolase with a pro-death activity from the protozoan parasite Leishmania major
Source: Cell Death Discov. 2019 May 24;5:99. doi: 10.1038/s41420-019-0178-2 (PMC6534544; doi:10.1038/s41420-019-0178-2)
Supplement: Supplementary file 2 — Supplemental table S1 [file 41420_2019_178_MOESM2_ESM.doc]

Table S1. Oligonucleotides used for RT-qPCR, overexpression, deletion, in situ C-terminal tagging or in situ N-terminal tagging of LmjF.36.6540.

| **Construction** | **Oligonucleotide name** | **Oligonucleotide sequence** |
| --- | --- | --- |
| RT-qPCR | dRTLm366540 | CCCAGTGTGAGTACAACCCC |
|  | rRTLm366540 | GGCAGCAAACCGAAGATGTC |
| *LmjF.36.6540* overexpression | dCLm366540pTH6 | GGGCAATTGATGTCCGCGGATCGCCC |
|  | rCLm366540pTH6 | GGGGTTAACATGGAGTGACTTCTTGAAGAA |
| *LmjF.36.6540* deletion | UpFLmjF366540 | AGTGGACTGCAGTATCTGAAGACGTTCCCCgtataatgcagacctgctgc |
|  | DownRLmjF366540 | CGCCGGCAGTCATACTGATGATGTGCACCGccaatttgagagacctgtgc |
|  | 5’gRNALmjF366540 | gaaattaatacgactcactataggGGGTGTCAAAAAAAAAGGTGgttttagagctagaaatagc |
|  | 3’gRNALmjF366540 | gaaattaatacgactcactataggCTGCTGGCGCGAAAACGTTGgttttagagctagaaatagc |
|  | sgRNA Reverse | AAAAGCACCGACTCGGTGCCACTTTTTCAAGTTGATAACGGACTAGCCTTATTTTAACTTGCTATTTCTAGCTCTAAAAC |
| LmjF.36.6540 *in situ* C-terminal mNeonGreen tagging | DownFLmjF366540 | GTGTTGGACTTCTTCAAGAAGTCACTCCATggttctggtagtggttccgg |
|  | DownRLmjF366540 | CGCCGGCAGTCATACTGATGATGTGCACCGccaatttgagagacctgtgc |
|  | 3’gRNALmjF366540 | gaaattaatacgactcactataggCTGCTGGCGCGAAAACGTTGgttttagagctagaaatagc |
|  | sgRNA Reverse Universel | AAAAGCACCGACTCGGTGCCACTTTTTCAAGTTGATAACGGACTAGCCTTATTTTAACTTGCTATTTCTAGCTCTAAAAC |
| LmjF.36.6540 *in situ* N-terminal mNeonGreen tagging | UpFLmjF366540 | AGTGGACTGCAGTATCTGAAGACGTTCCCCgtataatgcagacctgctgc |
|  | UpRLmjF366540 | GCAGCAGCGGTTCGGGCGATCCGCGGACATactacccgatcctgatccag |
|  | 5’gRNALmjF366540 | gaaattaatacgactcactataggGGGTGTCAAAAAAAAAGGTGgttttagagctagaaatagc |
|  | sgRNA Reverse Universel | AAAAGCACCGACTCGGTGCCACTTTTTCAAGTTGATAACGGACTAGCCTTATTTTAACTTGCTATTTCTAGCTCTAAAAC |
