# Supplementary figures and images for: A novel hydrolase with a pro-death activity from the protozoan parasite Leishmania major
Source: Cell Death Discov. 2019 May 24;5:99. doi: 10.1038/s41420-019-0178-2 (PMC6534544; doi:10.1038/s41420-019-0178-2)

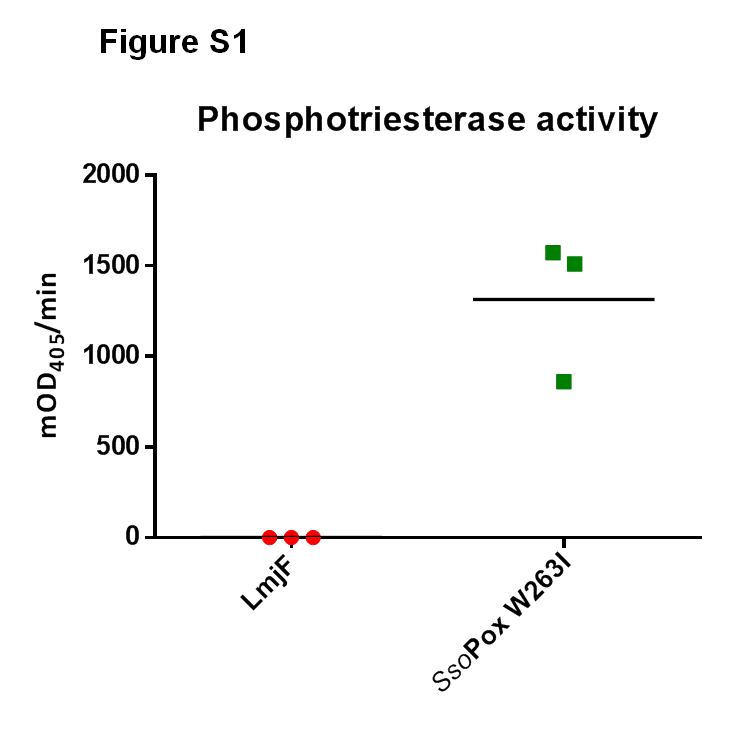

Supplement: Supplementary file 3 — Supplemental figure S1 [file 41420_2019_178_MOESM3_ESM.png]

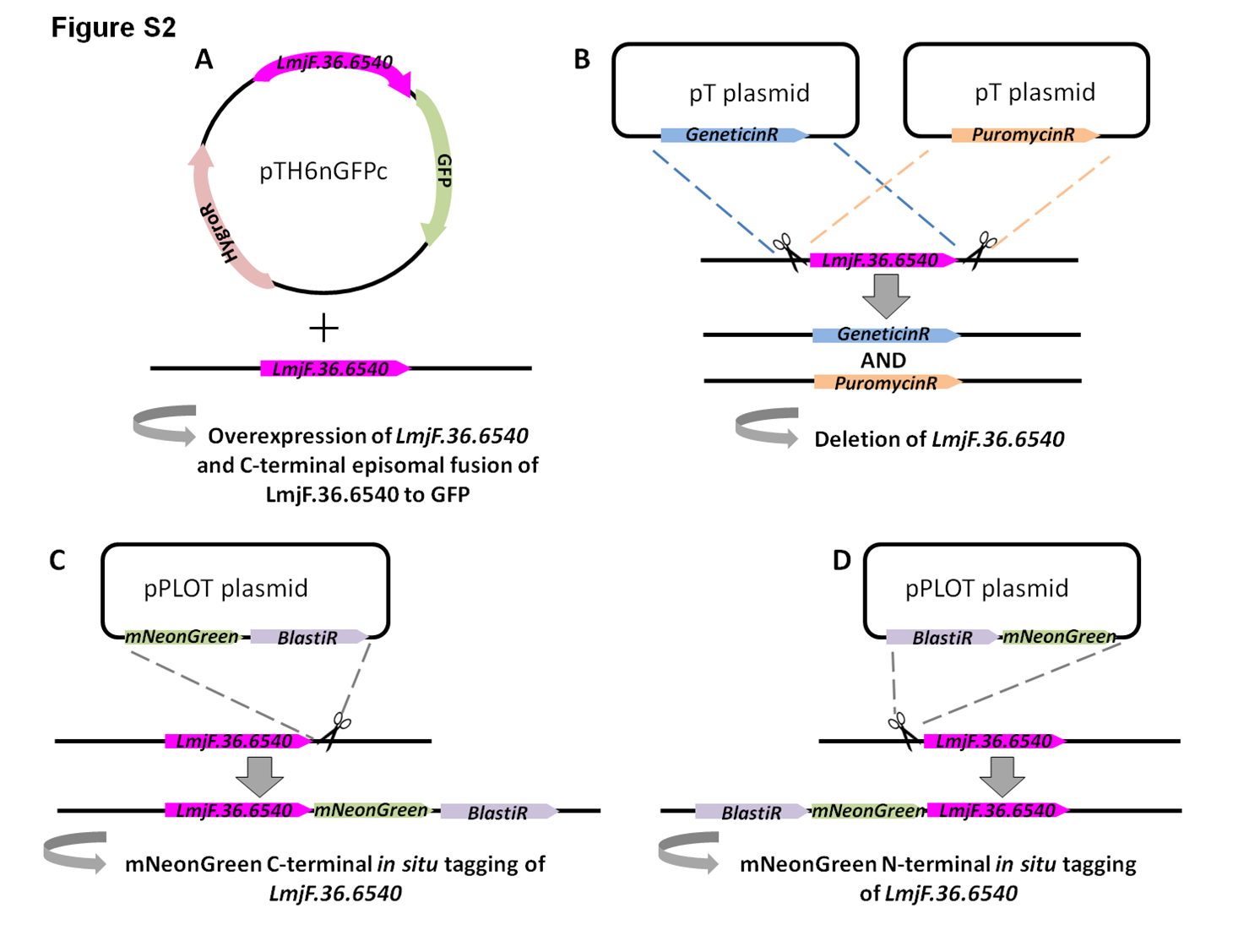

Supplement: Supplementary file 4 — Supplemental figure S2 [file 41420_2019_178_MOESM4_ESM.png]

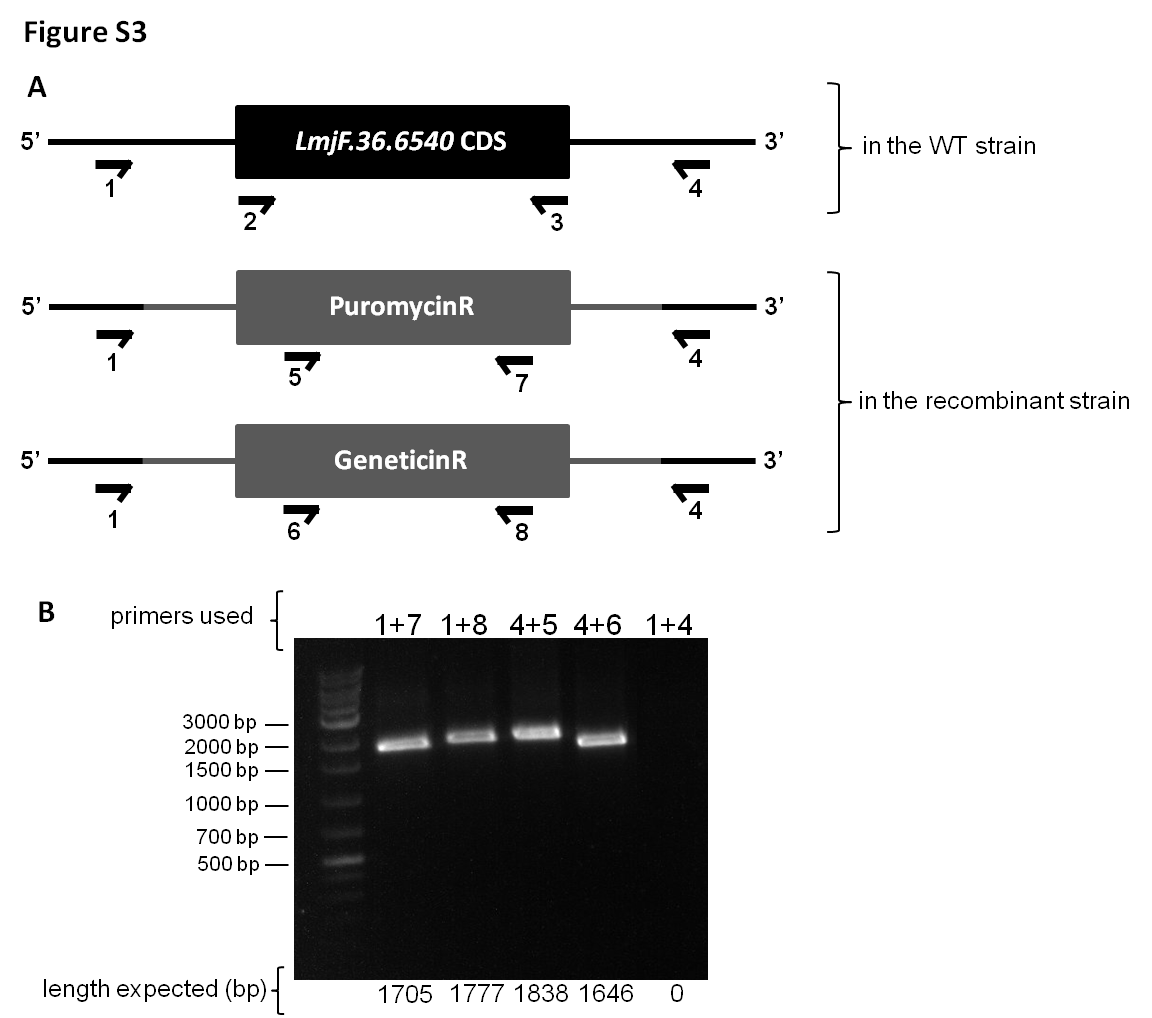

Supplement: Supplementary file 5 — Supplemental figure S3 [file 41420_2019_178_MOESM5_ESM.png]

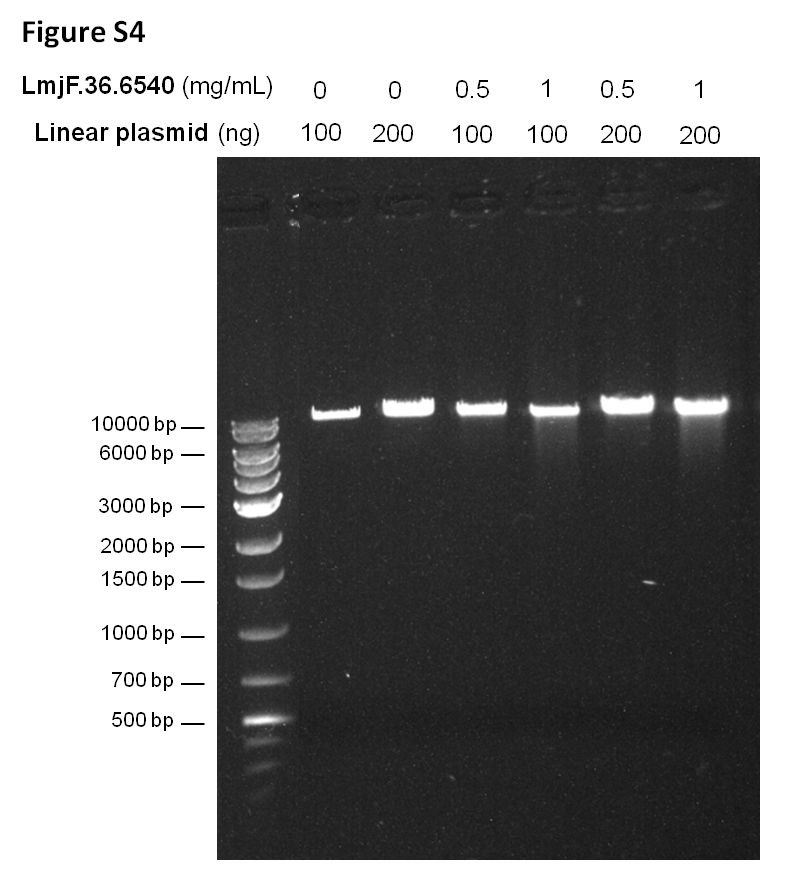

Supplement: Supplementary file 6 — Supplemental figure S4 [file 41420_2019_178_MOESM6_ESM.png]

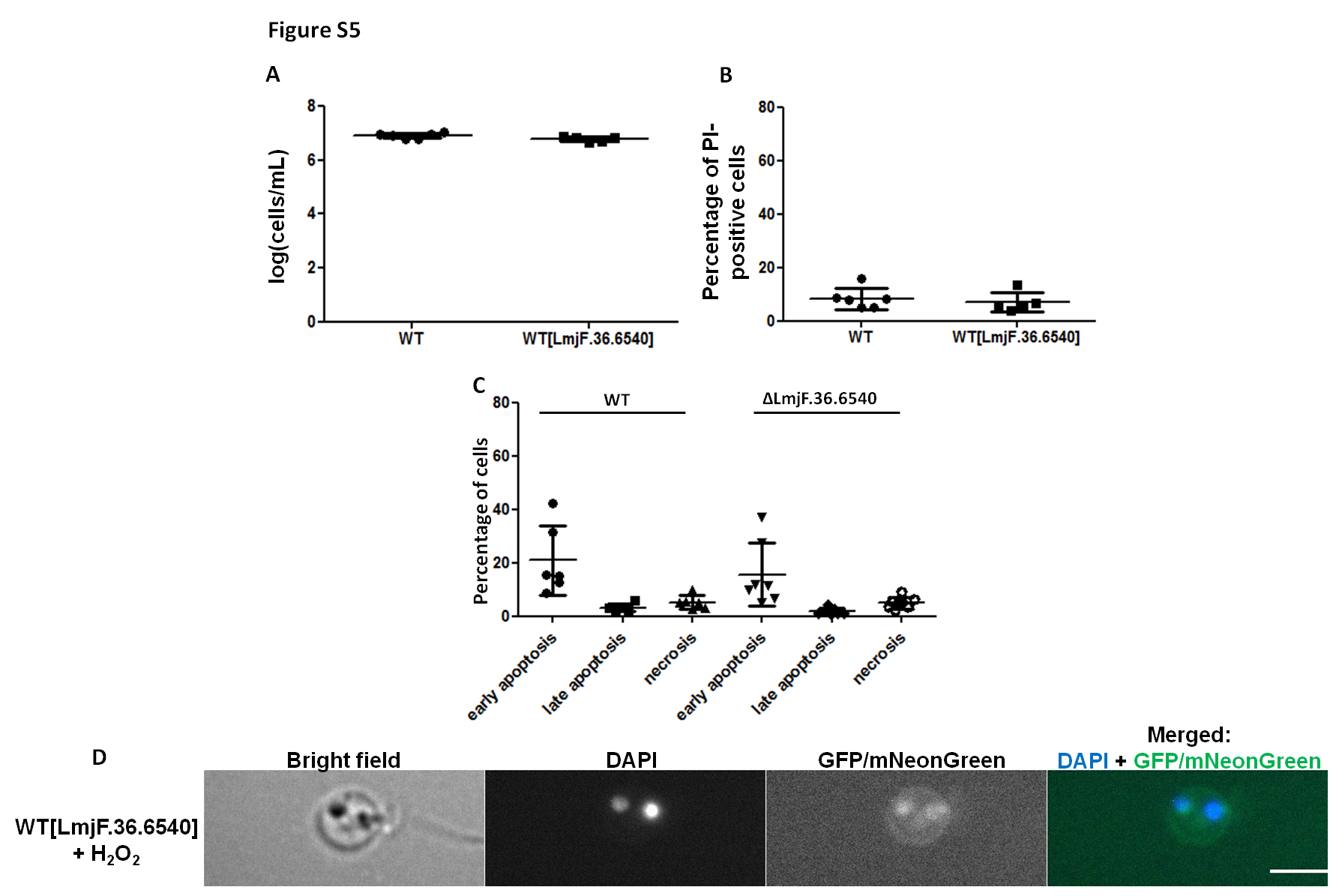

Supplement: Supplementary file 7 — Supplemental figure S5 [file 41420_2019_178_MOESM7_ESM.png]

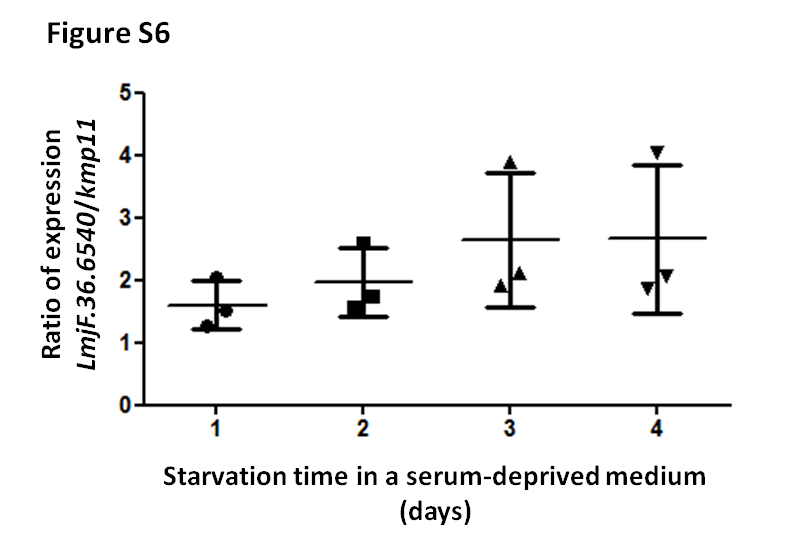

Supplement: Supplementary file 8 — Supplemental figure S6 [file 41420_2019_178_MOESM8_ESM.png]
